# Self-gated cardiac magnetic resonance perfusion imaging compared with X-ray angiography: a pilot study

**DOI:** 10.1186/1532-429X-14-S1-O39

**Published:** 2012-02-01

**Authors:** Alexis Harrison, Ganesh Adluru, Brent Wilson, Christopher McGann, Edward DiBella

**Affiliations:** 1 Cardiology, University of Utah, Salt Lake City, UT, USA; 2 Radiology, University of Utah, Salt Lake City, UT, USA; 3 UCAIR, University of Utah, Salt Lake City, UT, USA

## Background

Cardiovascular Magnetic Resonance (CMR) stress perfusion is an effective noninvasive diagnostic clinical tool when combined with late gadolinium enhancement imaging (LGE) for evaluation of ischemia, infarct, and cardiac prognosis. Good ECG-gating is essential to the commonly used perfusion sequences, but ECG-gating is problematic in obese patients, high field magnetic fields, and patients with arrhythmias. We describe a rapid radial-based self-gated (SG) perfusion acquisition for detection of perfusion defects and compare this to LGE and X-ray coronary angiography (XCA) in the detection of infarction or ischemia.

## Methods

In 5 patients (4 males, 1 female, 4 with paroxysmal atrial fibrillation, and 1 in atrial fibrillation during the CMR scan) with known or suspected obstructive coronary artery disease, a CMR study was performed including cine imaging, dynamic stress perfusion with adenosine, rest perfusion, and LGE. Gadoteridol (Prohance) 0.075 mmol/kg was injected for stress and subsequently rest perfusion and 230 time frames of 5 slices each were acquired over a minute (regardless of heartrate). A saturation recovery radial turboFLASH sequence was used without regard to the ECG. The images were reconstructed with an iterative compressed sensing method. Four out of the 5 patients had XCA. The SG perfusion images were qualitatively analyzed by 2 expert readers and correlated with LGE and XCA.

## Results

**Figure 1:**
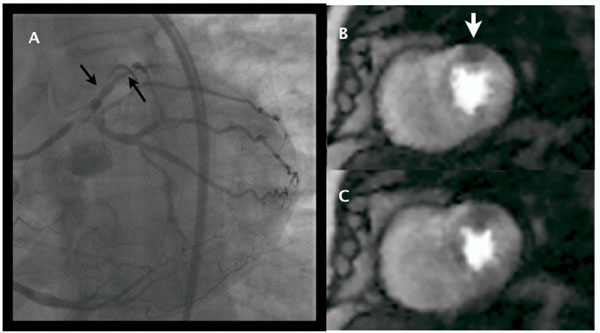
Coronary angiogram (A) and self-gated stress perfusion with images depicting diastolic and systolic cardiac phases (B, C). The stress perfusion shows a mid anterior wall defect (thick arrow). The angiogram shows severe in-stent stenosis in the first large diagonal and CTO of mid LAD (thin arrows).

**Figure 2:**
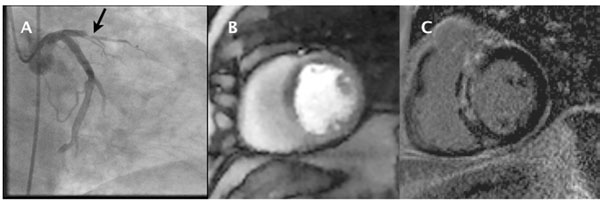
X-ray angiogram of STEMI (A) with 100% occlusion of the LAD and without any other significant flow-limiting coronary lesions. Post-PCI dynamic self-gating perfusion imaging showed a large anterior wall defect (B, rest not shown). LGE confirms a large anterior wall infarct resulting from the STEMI (C).

All SG perfusion acquisitions correlated with LGE and XCA in the detection of infarction or ischemia. Patient 1 had an anterior lateral SG stress only perfusion defect, no anterior lateral LGE, and a significant stenosis in a large D1 along with CTO of mid LAD on XCA (Fig. 1). Patients 2 and 3 were concordant for prominent scar on LGE and large SG stress and rest perfusion defects in the same area. One of these patients had XCA prior to the MRI that showed total LAD occlusion that was promptly reperfused and no other significant stenotic lesions (Fig. 2). Images in patients 4 and 5 were concordant with no perfusion defects, normal LGE, and normal coronary arteries on XCA. One of these patients was in atrial fibrillation during the scan and image quality was comparable to images taken in normal sinus rhythm.

## Conclusions

Self-gated radial-based dynamic MRI perfusion correlates with XCA in patients with ischemia, infarct, and normal coronary arteries. This technique may be particularly useful in patients with irregular heart rhythms such as atrial fibrillation where detection of ischemia is difficult. Future studies of SG sequences in a larger population of gating problematic patients are needed to determine sensitivity and specificity.

## Funding

No source.

